# The Potential Impact of a 20% Tax on Sugar-Sweetened Beverages on Obesity in South African Adults: A Mathematical Model

**DOI:** 10.1371/journal.pone.0105287

**Published:** 2014-08-19

**Authors:** Mercy Manyema, Lennert J. Veerman, Lumbwe Chola, Aviva Tugendhaft, Benn Sartorius, Demetre Labadarios, Karen J. Hofman

**Affiliations:** 1 School of Public Health, Faculty of Health Sciences, University of the Witwatersrand, Johannesburg, South Africa; 2 PRICELESS-SA, MRC/Wits Rural Public, Health and Health Transitions Research Unit, School of Public Health, University of the Witwatersrand, Johannesburg, South Africa; 3 School of Population Health, University of Queensland, Brisbane, Queensland, Australia; 4 Discipline of Public Health Medicine, School of Nursing and Public Health, University of KwaZulu-Natal, Durban, South Africa; 5 Population Health, Health Systems and Innovation (PHHSI), Human Sciences Research Council, Capetown, South Africa; 6 Johns Hopkins Bloomberg School of Public Health, Baltimore, Maryland, United States of America; The University of Tokyo, Japan

## Abstract

**Background/Objectives:**

The prevalence of obesity in South Africa has risen sharply, as has the consumption of sugar-sweetened beverages (SSBs). Research shows that consumption of SSBs leads to weight gain in both adults and children, and reducing SSBs will significantly impact the prevalence of obesity and its related diseases. We estimated the effect of a 20% tax on SSBs on the prevalence of and obesity among adults in South Africa.

**Methods:**

A mathematical simulation model was constructed to estimate the effect of a 20% SSB tax on the prevalence of obesity. We used consumption data from the 2012 SA National Health and Nutrition Examination Survey and a previous meta-analysis of studies on own- and cross-price elasticities of SSBs to estimate the shift in daily energy consumption expected of increased prices of SSBs, and energy balance equations to estimate shifts in body mass index. The population distribution of BMI by age and sex was modelled by fitting measured data from the SA National Income Dynamics Survey 2012 to the lognormal distribution and shifting the mean values. Uncertainty was assessed with Monte Carlo simulations.

**Results:**

A 20% tax is predicted to reduce energy intake by about 36kJ per day (95% CI: 9-68kJ). Obesity is projected to reduce by 3.8% (95% CI: 0.6%–7.1%) in men and 2.4% (95% CI: 0.4%–4.4%) in women. The number of obese adults would decrease by over 220 000 (95% CI: 24 197–411 759).

**Conclusions:**

Taxing SSBs could impact the burden of obesity in South Africa particularly in young adults, as one component of a multi-faceted effort to prevent obesity.

## Introduction

Globally, overweight (25≤BMI<30) and obesity (BMI≥30) have reached epidemic proportions. In 2008, 1.46 billion adults worldwide were either overweight or obese and this figure is expected to increase to 3.28 billion by 2030 [1], [2]. In low and middle-income countries (LMICs), including South Africa (SA), overweight and obesity also continue to rise [3]. The 2003 SA Demographic and Health Survey and the 2012 SA National Health and Nutrition Examination Survey (SANHANES-1) show that in less than a decade, obesity prevalence has increased from 8.8% to 10.6% in men and from 27.4% to 39.2% in women [4], [5]. The 2013 Global Burden of Disease (GBoD) Study reports that the prevalence increased to 13.5% and 42.0% for men and women respectively in 2013 [6].

The association of obesity with hypertension and non-communicable diseases (NCDs) such as diabetes, stroke and cardiovascular disease (CVD) is well-established and the risk of these NCDs increases with increasing body mass [2], [7], [8]. The World Health Organisation estimates that world-wide, obesity-related diseases account for over 2.8 million deaths annually [9]. The 2010 GBoD Study shows a substantial shift in the burden of disease from communicable disease to NCDs. In 1990, 47% of DALYs worldwide were from communicable, maternal, neonatal, and nutritional disorders, 43% from NCDs, and 10% from injuries. By 2010, these were 35%, 54%, and 11%, respectively [10]. In SA, 7% of all deaths and 2.9% of all disability-adjusted life years (DALYs) were attributable to excess body weight (BMI≥21kg/m^2^) in 2000 [11]. In 2004, 28% all DALYs were attributable to NCDs [11], [12]. Analysis of the GBoD data for South Africa shows that in 2010 29.2% of all DALYs were attributable to NCDs.

Obesity has significant direct and indirect economic costs. In Europe and the United States of America (USA), increased body mass is associated with an increase in healthcare costs [13]–[15]. In SA, moderate obesity (BMI 30–35 kg/m^2^) is associated with an 11% increase in healthcare costs and severe obesity (BMI >35 kg/m^2^) with a 23% increase [16]. In 1991 the annual cost of CVD in SA was 4·1 to 5·0 billion Rands or 17.4 to 21.3 billion Rands at 2013 prices, equivalent to 1.6 to 2.0 billion US dollars (USD) using 2014 conversion rates [17].

Observational and experimental studies show that consumption of SSBs promotes weight gain in adults and children [18], [19]. SSB consumption has increased across the globe and time-trend data over the past three decades show a close parallel with the escalating obesity epidemic [20], [21]. SSBs are thought to lead to weight gain due to their high sugar content and incomplete compensation for total energy at subsequent meals following intake of liquid calories [20], [21]. A 330 ml can of carbonated soft drink contains about 40 g of sugar and sweetened fruit juice close to 45 g of sugar. Although further research is still needed, there is sufficient evidence to indicate that reducing SSB consumption will impact obesity prevalence. [18], [19], [22], [23]. There is also strong evidence that this would impact diseases associated with SSB consumption such as dental caries, CVD, type 2 diabetes mellitus and metabolic syndrome [20], [21], [24]–[26].

Evidence from modelling and meta-analysis studies in the United Kingdom (UK), Ireland, India and Brazil shows that fiscal policy, such as taxing SSBs, may reduce SSB consumption and consequently total energy intake, leading to reductions in population weight [27]–[31]. The Danish, French, Hungarian and Mexican governments have implemented an SSB tax.

In Denmark the tax was passed in the 1930s but repealed in 2013 in a bid to ‘create jobs and boost the economy’ [32]. Hungary and France implemented SSB taxes in 2011 and 2012 respectively [33], [34]. These taxes have attracted resistance from industry, with the Union of European Soft Drinks Associations maintaining that they are regressive and have not proved to be effective [35]. In Mexico an SSB tax was passed in 2013 amid opposition from industry, economists and opposition political parties [36]. Evidence to inform a similar policy in SA is not yet available.

The SA Department of Health (DOH) has demonstrated willingness to use fiscal and regulatory policy tools for improving public health. Increases in tobacco excise tax and tobacco control regulations decreased aggregate cigarette consumption by a third between 1993 and 2003, and per capita consumption by 40%. The instrument with the biggest impact was excise taxation [37]. In 2011 regulations were passed to limit the level of trans-fatty acids in food to a maximum of 2% of the oil content [38], [39]. In 2013, further regulations to limit salt content for various foodstuffs including bread, butter, breakfast cereals, ready-to-eat snacks, processed meat and soup powder were passed [40]. The DOH Strategic Plan for the Prevention and Control of NCDs 2013–17 lists taxes on foods high in sugar as potentially cost-effective strategies and “best buys” for addressing diet and obesity [39]. In line with these plans, the Minister of Health has recently called for regulations on foods high in sugar [41].

The aim of this study is to model the effect of a 20% SSB tax on the prevalence of obesity in SA adults. It will provide evidence on the potential impact of fiscal policy on SSB consumption and obesity in SA and enable the DOH to consider this as a lever to prevent and reduce the burden of disease resulting from obesity-related NCDs.

## Materials and Methods

### Overview of model

We constructed a mathematical simulation model to estimate the effect of a 20% SSB tax on the prevalence of obesity, using 2012 as the baseline year.

The model compares two populations: an unchanged reference population and a counterfactual intervention population in which changes in SSB price are translated into changes in SSB consumption and subsequently body mass change. Previously published price elasticities were used to estimate the effect of the tax on SSB consumption.

**Figure 1:**
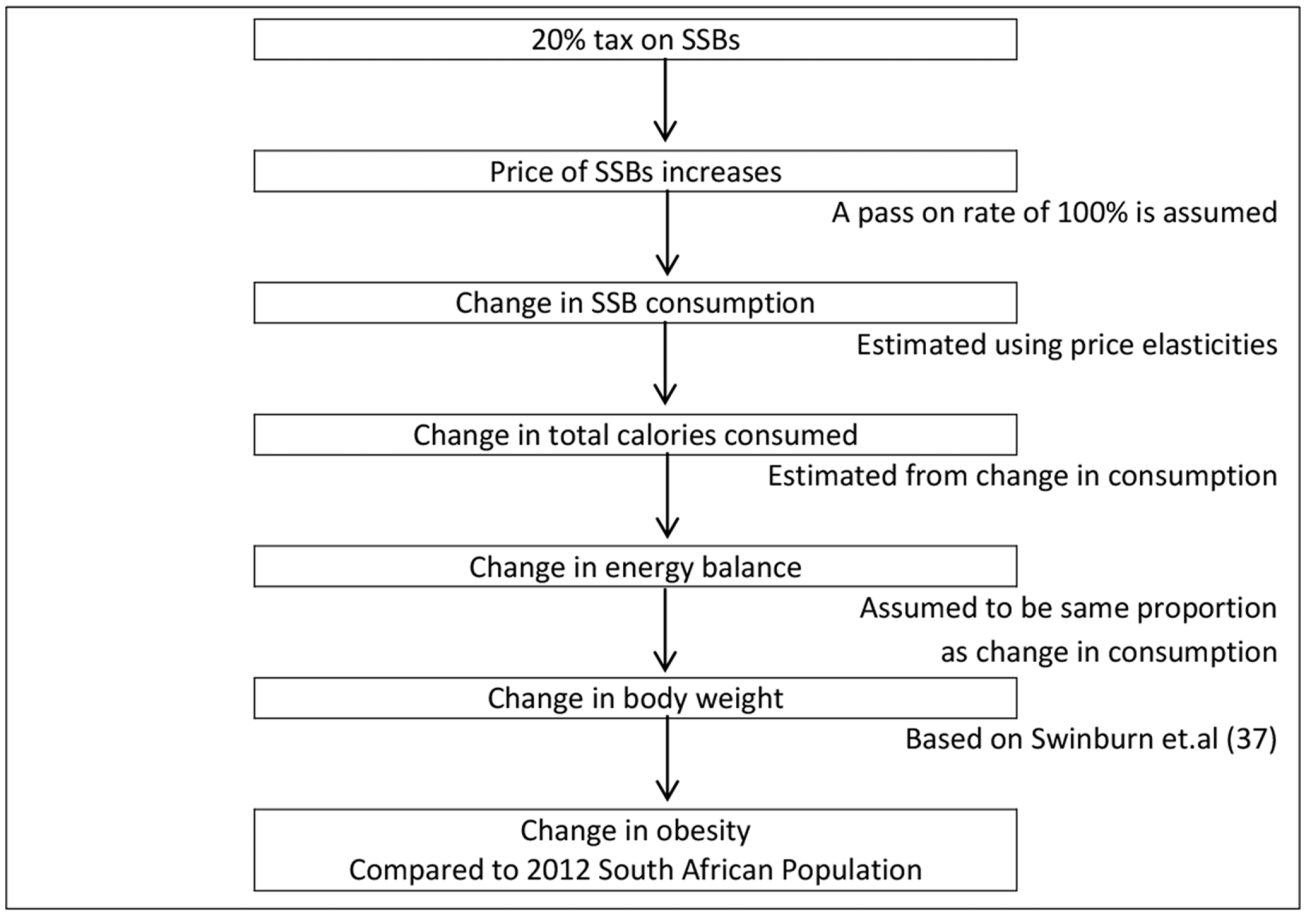
Analytical framework for the effect of a 20% tax on obesity in South Africa. Figure 1 is the hypothesised causal pathway of the effect of a 20% tax on SSBs. The assumptions are indicated in the text outside of the text boxes. The arrows show how each step of the pathway leads to the next.

Published equations linking energy intake to body weight were used to estimate changes in body mass index and obesity [42]. The model was implemented in Microsoft Excel (2010). Similar models have been used previously to estimate the effects of tax and other interventions on obesity prevalence [27], [28], [43], [44]. Figure 1 shows the analytical framework for the model, which has been adapted from work done by Briggs et al. and Sacks et al. in estimating the effect of interventions on obesity [28], [45].

### Data and assumptions

#### Price elasticities

Price elasticity measures the rate of response of the quantity of a good demanded when price increases. Own-price elasticity refers to the change in demand that occurs for a good in response to price changes of the same good. Cross-price elasticity is the change in purchases that occur for a good in response to price changes of another good. We used price elasticity estimates from a systematic review and meta-analysis of studies done in the USA, France, Mexico and Brazil to derive the changes in energy intake [46].

#### Pass on rate

SSB tax may be passed on in full to consumers, or manufacturers and retailers may absorb some of the tax by reducing tax margins. An Irish study by Briggs et al. assessing the impact of a 10% SSB tax on obesity assumed a pass on rate of 80–100% [47], based on evidence from France that the pass on rate for carbonated drinks is 100%, 60% for fruit drinks and 85% for flavoured water [48]. Evidence from the USA shows that alcohol taxes are passed on in full [49]. For this study we assumed a 100% pass on rate.

#### Prevalence of obesity in SA

BMI estimates were obtained from anonymised datasets of Wave 3 Version 1 of the National Income Dynamics Study (NIDS) [50]. NIDS, SA's first national panel study, was implemented by the Southern Africa Labour and Development Research Unit based at the University of Cape Town's School of Economics. A total of 28 255 individuals in 7 305 households were recruited in the base wave in 2008 [51]. Waves 2 and 3 were conducted in 2010/2011 and 2012 respectively [52], [53]. Stratified, two-stage cluster sampling was used to sample the households in Wave 1 and all resident members of private households at selected dwelling units were eligible to participate [51], [54]. Data were collected through household and individual questionnaires and anthropometric measurements [55].

Adult men and women aged 15 years and above with a valid height and weight measurement were included in our analysis. We cleaned and coded the data in STATA Version 12.1 (College Station, TX) [56]. The STATA *svyset* command was used to account for design effect. BMI was computed as weight in kilograms divided by the square of height in metres. BMI values falling below and above the 1^st^ and 99^th^ percentiles were excluded from the analysis.

Data were fitted to the log-normal distribution in Microsoft Excel (2010) using the least squares method. Polynomial functions were used to fit the means and standard deviation across age-groups, separately for men and women. The lognormal or gamma distribution can be used for positively skewed data. We compared the two and found no significant difference between the results. The log-normal distribution however had better fitting properties and it has previously been used to model population BMI in similar modelling studies [43], [57], [58]. The fitting procedure and comparison between the two distributions is described in Text S1. Figure S1 and Figure S2 present a comparison of the raw and log-normal fitted BMI data and a counterfactual distribution in which the mean BMI was increased by five BMI points. Table S1 shows the results of the comparison between the gamma and lognormal distribution for fitting the BMI data.

#### Consumption estimates

An SSB was defined as a non-alcoholic drink with added sugar and this comprised carbonated sweetened drinks, sweetened fruit juices and squash concentrates. Data from the SANHANES-1 were used to estimate SSB, milk and unsweetened fruit juice consumption in adults aged 15 years and above [5]. The SANHANES-1, a baseline cross-sectional survey of the SANHANES, obtained questionnaire-based data through interviews in combination with some health measurements. Multi-stage disproportionate, stratified cluster sampling was used to select the study population and all individuals residing at the selected households were eligible to participate.

Participants were asked the number of cups of milk and unsweetened juice they drank per day with response categories being more than two cups, one to two cups, half to one cup, less than one cup, none and don't know.

One cup was assumed to be 250 ml, the standard size of a cup. In our analysis the mid-point of each range was taken as the average. Those who consumed more than two cups were assumed to have taken 500 ml because they would have drunk at least two cups of drink and those who consumed less than half a cup were assumed to have taken 125 ml because they would have drunk at most half a cup of drink. This capping was done to enable calculation of the average consumption of milk and unsweetened fruit juice per person per day.

For SSBs the participants were asked the frequency of consumption in the last week with response options none, every day, one to three times last week and four to six times last week. We assumed one serving to be 330 ml, the size of a can of carbonated soft drink and that one serving was drunk per day. The mid-point of each frequency of consumption category was taken as the average consumption for the category. Based on SAB Miller figures, diet drink consumption was estimated as 4% of SSB consumption [59].

#### Population estimates

The baseline population estimates by sex and five-year age-groups were taken from the Statistics SA 2012/2013 Release [60].

### Modelling

#### Step 1 - Change in SSB consumption

A 20% tax with a 100% pass on rate was used to estimate a price rise, which together with price elasticities was used to estimate the percentage change in purchasing and hence consumption of SSBs. Consumption of SSBs was in litres per day per person, calculated per 5-year age bands. An own-price elasticity of −1.299 was used for SSBs, meaning that a 10% increase in the price of SSBs would lead to a decrease in consumption of 13%.

The cross-price elasticities of milk, fruit juice and diet drinks were 0.129, 0.388 and −0.423 respectively (i.e. a 10% SSB price increase would increase milk and fruit juice consumption by 1.2% and 3.8% respectively, decrease diet drink consumption by 4.2%) [46]. The same price elasticities were applied across all age and sex categories.

#### Step 2 – Change in energy intake

We used average calorie density estimates for each drink to convert change in volume consumed to change in energy intake, assuming the percentage change in energy intake to be the same as percentage change in volume consumed. SSBs were assumed to have an energy density of 1800 kilojoules (kJ)/litre (average of Coca Cola SSBs), 1340 kJ/l for unsweetened juice, 2540 kJ/l for whole milk [61] and 4 kJ/l for diet drinks. We assumed that all milk drunk was full cream milk. The changes in caloric intake for each beverage type were summed to give the net change in energy intake.

#### Step 3 – Change in body mass index and obesity prevalence

Change in body mass was estimated using equations published by Swinburn et al. al [42], which state that a daily increase in energy intake of 94 kJ/day is needed for a change in body weight of 1 kg in equilibrium for adults. The change in average body mass was converted to change in average BMI by applying average height estimates for each age-group, derived from the NIDS Wave 3 data set. We applied Rose's theorem that the mean predicts the number of deviant individuals to estimate the prevalence of obesity from the mean BMI for each age-group and sex [62].

### Uncertainty analysis

Ninety-five percent uncertainty intervals were estimated using Monte Carlo simulation using the Ersatz programme (Barendregt JJ, Brisbane 2007), varying the own-and cross-price elasticity estimates [30], the conversion factor between energy consumption change and weight change [42], and the consumption estimates by age and sex for all four beverages. The estimates, distributions and sources of the uncertainty inputs are presented in Tables 1 and 2.

**Table 1:** Uncertainty distributions and sources of model parameters: price elasticities and energy intake conversion factor.

Parameter	Mean Value(SD)	Uncertainty distribution	Sources and assumptions
SSB own price elasticity	−1.30 (0.11)	Normal	Review and meta-analysis, Cabrera et al (25)
Milk cross-price elasticity	0.13 (0.1)	Normal	Review and meta-analysis, Cabrera et al (25)
Fruit juice cross-price elasticity	0.39 (0.19)	Normal	Review and meta-analysis, Cabrera et al (25)
Diet drinks cross-price elasticity	−0.42 (0.10)	Normal	Review and meta-analysis, Cabrera et al (25)
Daily energy intake required for 1 kg change in weight (kJ/kg/day)	94 (2.96)	Normal	Swinburn et. al (37)

**Table 2:** Uncertainty distributions and sources of model parameters: Consumption estimates in l/person/day: Mean value (SD) Distribution: Normal; Source: SANHANES-1.

Age	SSB	Milk	Unsweetened Fruit juice	Diet drinks
15–24	0.21 (0.01)	0.21(0.01)	0.21 (0.01)	0.01 (5.58×10^−4^)
25–34	0.20 (0.02)	0.20 (0.01)	0.20 (0.01)	0.01 (6.11×10^−4^)
35–44	0.18 (0.01)	0.21 (0.01)	0.21 (0.01)	0.01 (5.91×10^−4^)
45–54	0.17 (0.01)	0.21 (0.02)	0.20 (0.02)	0.01 (4.50×10^−4^)
55–64	0.15 (0.02)	0.19 (0.02)	0.17 (0.02)	0.01 (8.27×10^−4^)
65+	0.12 (0.02)	0.22 (0.02)	0.17 (0.02)	4.80×10^−3^ (6.73×10^−4^)

### Sensitivity analysis

Deterministic two-way sensitivity analysis was performed to assess the effect on the prevalence of obesity of varying the tax and pass on rates. A range of 10%–30% was used for the level of tax and 80–120% for the pass on rate. The tax rate and pass on rate were chosen for sensitivity analysis due to their uncertainty and lack of locally applicable empirical evidence on the best estimate. One-way sensitivity analysis was also performed for the unit volume of a serving of SSB due to there being several serving sizes in which SSBs are available e.g. 200 ml, 250 ml, 330–350 ml and 500 ml.

### Ethics

This study was based on secondary analysis of human participant data collected through two national surveys, the SANHANES-1 for baseline consumption data and NIDS for baseline prevalence of obesity. Ethics approval was not sought for because the NIDS dataset used in this study is an edited, anonymised dataset for public distribution available through DataFirst, a data service based at the University Of Cape Town, South Africa.

The data set contains only a limited number of variables in order to protect the identities of the NIDS respondents and is publicly available from the DataFirst data portal [63]. The SANHANES-1 consumption data was obtained with no identifying information. The two national surveys themselves independently obtained ethics approval before they commenced [5], [51].

## Results

### Baseline consumption and energy intake from drinks

The SANHANES-1 data show that on average SA adults consume 184 ml of SSBs, 200 ml of unsweetened fruit juice and 204 ml of milk a day. Consumption of SSBs at baseline showed a declining trend as age increased, with the 15–24 age-group drinking the highest amount and those aged 65 and over drinking the least (Table 3).

**Table 3:** Daily consumption of different drinks at baseline.

Daily consumption of drinks in ml (95% confidence intervals)				
Age	SSB	Fruit Juice	Milk	Diet Drinks
**15–24**	209 (183–238)	211 (189–236)	205 (182–232)	8 (7–10)
**25–34**	199 (171–231)	205 (179–233)	199 (197–227)	8 (7–9)
**35–44**	181 (154–212)	210 (182–241)	205 (179–235)	7 (6–9)
**45–54**	171 (144–188)	193 (159–233)	211 (180–246)	7 (6–8)
**55–64**	149 (119–200)	171 (142–207)	195 (161–235)	6 (5–8)
**65+**	120 (92–158)	166 (132–208)	217 (174–270)	5 (4–6)

### Change in daily energy intake and BMI

**Figure 2:**
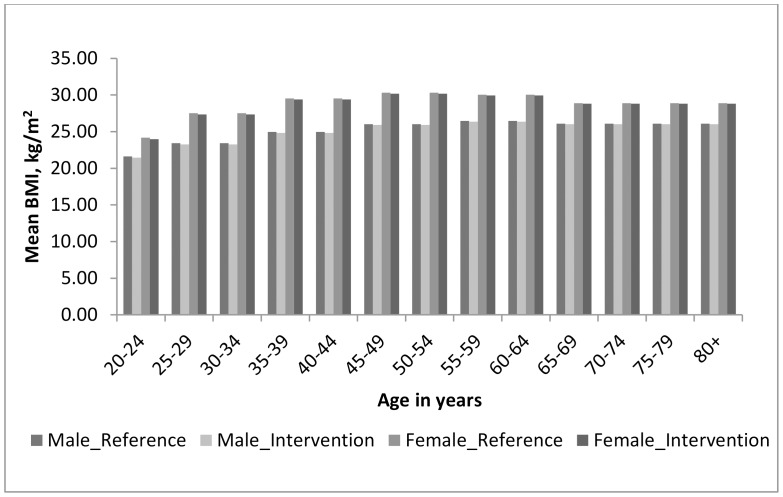
Mean BMI before and after the intervention by age and sex. Mean BMI of the reference population (Male_reference; Female_reference) at baseline and the intervention population (Male_intervention; Female_intervention) after the 20% tax intervention for males and females, for each age-group.

Table 4 presents the impact of the tax on total daily energy intake and BMI. The average reduction in energy intake is 36.0 kJ per person per day. There is marked variation in the changes in both energy intake and BMI by age, with the largest and most significant reductions in the younger age-groups. Figure 2 shows the mean BMI levels before and after the intervention for both men and women. The mean BMI for women remained above 25 kg/m^2^ after the intervention for all age-groups except for those aged 20 to 24 years. Energy intake and BMI changes are largely not statistically significant in those aged 60 and above.

**Table 4:** Estimated change in energy intake and body weight after 20% tax.

Age	Change in average energy intake in kJ/person/day (95% uncertainty intervals)	Change in mean BMI in kg/m^2^ (95% uncertainty intervals)	
	All	Male	Female
**20–24**	−45.78 (−80.14, −12.04)	−0.17 (−0.32, −0.04)	−0.19 (−0.35, −0.05)
**25–29**	−43.28 (−75.71, −9.64)	−0.16 (−0.29, −0.03)	−0.18 (−0.32, −0.04)
**30–34**	−43.02 (−74.37, −9.55)	−0.16 (−0.28, −0.03)	−0.18 (−0.32, −0.04)
**35–39**	−35.48 (−67.73, −0.89)	−0.13 (−0.26, 0.00)	−0.15 (−0.29, 0.00)
**40–44**	−35.45 (−68.67, −1.80)	−0.13 (−0.26, −0.01)	−0.15 (−0.28, −0.01)
**45–49**	−33.04 (−63.36, −0.15)	−0.12 (−0.24, 0.00)	−0.14 (−0.27, 0.00)
**50–54**	−33.03 (−64.07, −0.85)	−0.12 (−0.24, 0.00)	−0.14 (−0.27, 0.00)
**55–59**	−27.73 (−57.71, 3.65)	−0.10 (−0.22, 0.01)	−0.12 (−0.25, 0.02)
**60–64**	−27.93 (−58.14, 4.07)	−0.10 (−0.22, 0.01)	−0.12 (−0.25, 0.02)
**65–69**	−15.98 (−45.74, 15.56)	−0.06 (−0.17, 0.06)	−0.07 (−0.20, 0.07)
**70–74**	−16.03 (−47.13, 16.83)	−0.06 (−0.18, 0.06)	−0.07 (−0.20, 0.07)
**75–79**	−16.23 (−46.99, 16.43)	−0.06 (−0.18, 0.06)	−0.07 (−0.20, 0.07)
**80+**	−15.78 (−45.16, 15.29)	−0.06 (−0.18, 0.06)	−0.07 (−0.19, 0.07)

### Effect on obesity

The prevalence of obesity is projected to go down by 3.8% (95% CI: 0.4%–7.2%) in men, equating to a 0.5 percentage point change and 2.4% (95% CI: 0.3%–4.4%) in women, equivalent to a 0.8 percentage point change.

**Figure 3:**
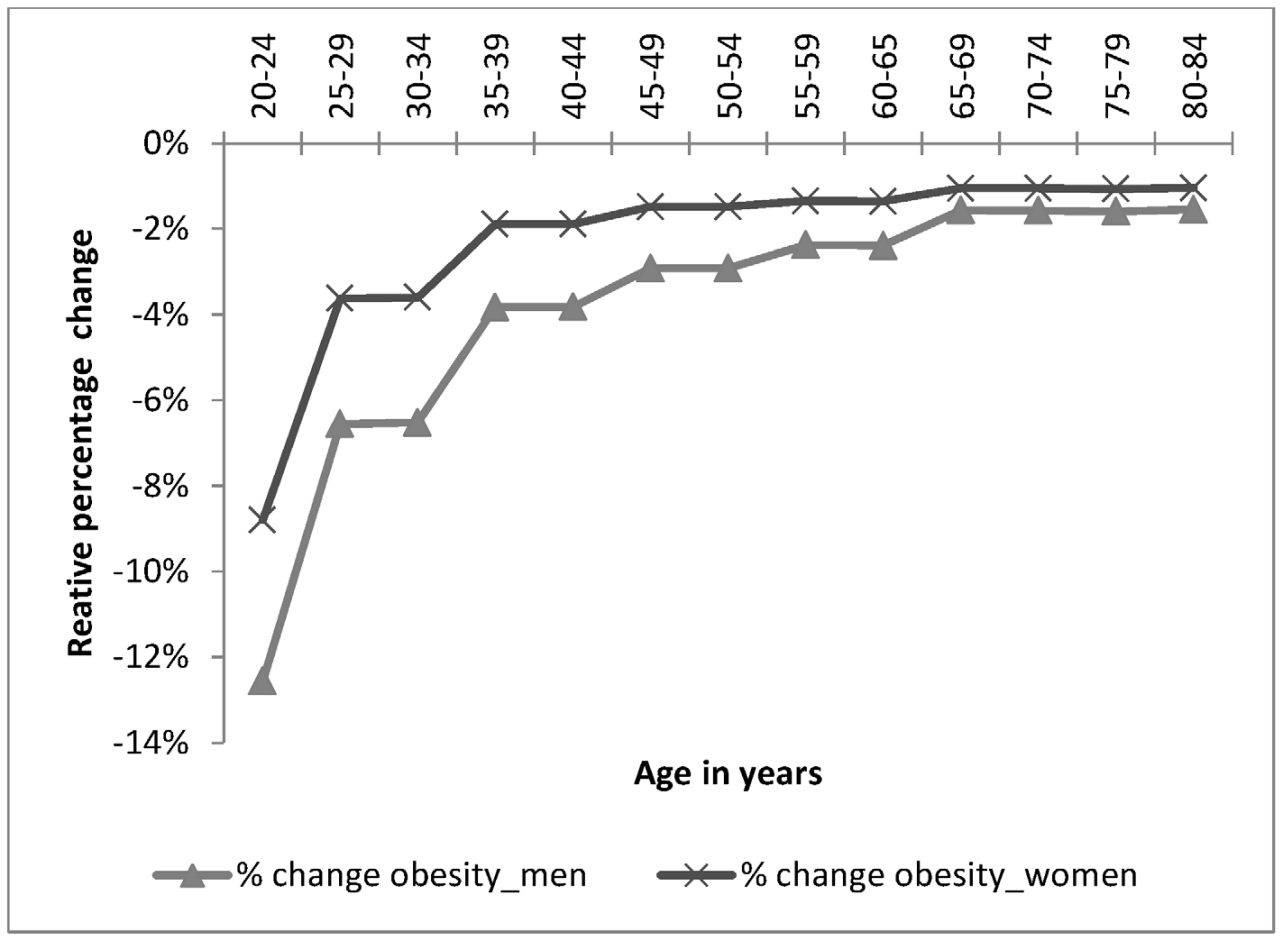
Percentage change in obesity after the intervention. The graph shows the estimated relative percentage changes in obesity in men (% change obesity_male) and women (% change obesity_female), by age as a result of the 20% SSB tax.

The number of obese women is expected to decrease by 142 217 (16 550–265 039), and obese men by 80 452 (16 060–147 284). Figure 3 shows the relative percentage change in obesity by age and sex. Overall, a greater relative decrease in the prevalence of obesity was observed in men than in women although mean MI shifts were on average greater in the women than the men. This is because the baseline mean BMI for women was higher than men for all age groups.

### Sensitivity analysis

**Figure 4:**
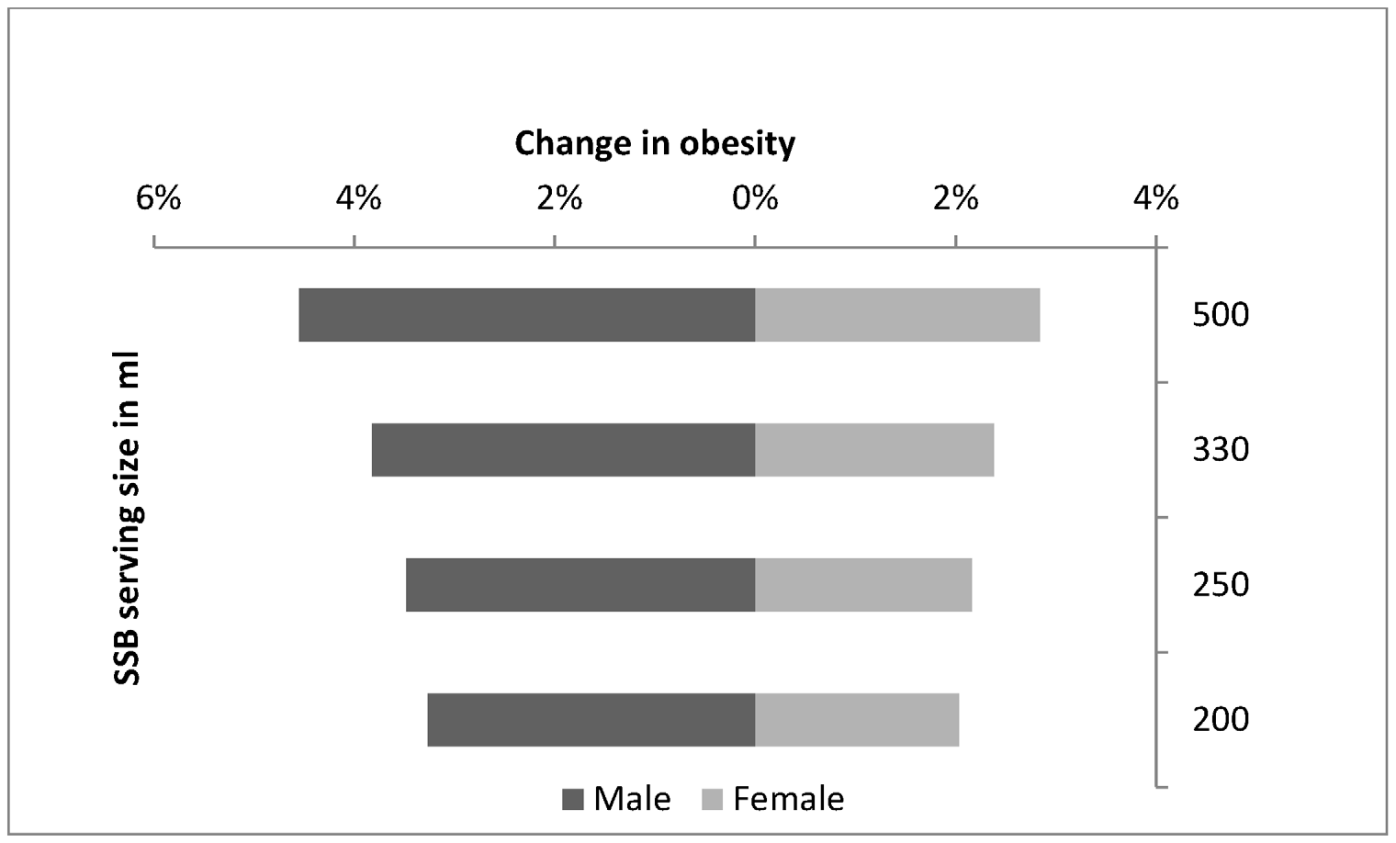
Tornado plot for the sensitivity analysis of the volume of an SSB serving. Tornado plot showing sensitivity analysis of the corresponding change in obesity due to different unit sizes of an SSB serving. Unit sizes tested: 200 ml, 250 ml, 330 ml and 500 ml.

The prevalence of obesity at different tax levels and pass on rates was compared. The results (Table 5) show that greater reductions in obesity prevalence resulted from increasing the tax even when the pass on rate is less than 100%. Figure 4 is a tornado plot showing results of the sensitivity analysis for the SSB serving size. Smaller SSB serving sizes resulted in less relative change in the prevalence of obesity. The greatest difference was 0.6 percentage points between a 200 ml and 330 ml portion and a 500 ml and 330 ml portion in men. The men appear to be affected more by changes in SSB unit size than the women.

**Table 5:** Two-way sensitivity analysis of the effect of changing pass on rate and tax rate on obesity.

Change in obesity Men					
	Pass on rate				
**Tax rate**	80%	90%	100%^1^	110%	120%
**10%**	−1.9%	−2.1%	−2.3%	−2.4%	−2.6%
**20%** 1	−3.3%	−3.6%	−3.8%	−4.1%	−4.3%
**30%**	−4.3%	−4.6%	−4.9%	−5.2%	−5.4%
**Women**					
**10%**	−1.1%	−1.3%	−1.4%	−1.5%	−1.6%
**20%** 1	−2.0%	−2.2%	−2.4%	−2.5%	−2.7%
**30%**	−2.7%	−2.9%	−3.1%	−3.3%	−3.4%

1 Value used in model.

## Discussion

A 20% SSB tax in SA is predicted to a reduce obesity by 3.8% in adult males and 2.4% in females. The average reduction in energy intake is estimated to be 30.0 kJ per person per day. The younger age-groups are the biggest consumers of SSBs and may benefit most from an SSB tax.

Our model and results are comparable to other studies showing the effect of taxing SSBs on obesity. The study by Briggs et al. estimated that a 10% SSB tax may result in an overall reduction in obesity of 1.2% in men and 1.3% in women [28].

A similar study in the UK modelled the effect of a 20% tax and estimated an overall reduction of 1.3% [27]. In India a 20% tax was estimated to reduce obesity by 3% [31].

### Study strengths and limitations

This is the first SA study to quantify the potential effect of tax on SSB consumption and obesity. There is a dearth of literature on this type of intervention in LMICs where the obesity prevalence and NCDs are increasing [6]. Similar studies were conducted in Brazil and India [29], [31] but none have been done in Africa.

This study has several strengths. Firstly, we used nationally representative SA data to estimate baseline consumption of different drinks and baseline prevalence of obesity. This increases the generalizability of our results. In addition, the height and weight from the survey were anthropometrically measured, which should allow for more accurate BMI estimates. Secondly, the model accounts for substitution of SSBs with other drinks through the use of cross-price elasticities. This prevents the overestimation of the reduction in total liquid caloric consumption. Thirdly, the model demonstrates the differential effects of the tax by age and sex.

A limitation of this study is the lack of SA-specific own- and cross-price elasticity data. The estimates used were pooled results derived from a systematic review and meta-analysis [30]. Our estimates were close to those used in previous work in Europe and the USA [27], [28], [44]. Variations in the price elasticities would have potentially impacted our results in several ways. A lower own-price elasticity, such as was found by Basu et.al [31] would have led to smaller changes in SSB consumption and subsequently more modest changes in obesity. Briggs et al [27] found the cross-price effect for diet drinks to be in the opposite direction to the effect used in our model, 0.304 for concentrated SSBs to concentrated diet drinks and 0.167 for non-concentrated SSBs to non-concentrated diet drinks, compared to −0.423. An increased demand for diet drinks would, however, have minimal impact due to the low energy content of those drinks. Both studies quoted above used smaller fruit juice cross-price elasticities than were used in our study.

Using similar values in our model would have led to lower energy intake estimates than reported and hence more reductions in obesity.

Our model did not include the substitution effect of other sweetened drinks such as coffee, tea and hot chocolate, and other non-sweetened drinks like water. Other studies have shown that the demand for tea and coffee, as well as water goes up with SSB price increases [27], [31]. We did not account for the differential effects in price elasticities between carbonated SSBs, drinks from concentrates, and sweetened fruit drinks due to the unavailability of this data. Briggs et.al found that an increase in the price of non-concentrated SSBs resulted in increased demand for concentrated SSBs and vice versa [27]. We also assumed similar price elasticities for both men and women, and for all age and income groups. Evidence from some studies suggests that demand for SSBs would decrease the most in those in the lowest income group [28], [31], [64], some studies found the effects similar across income strata [65], and others found that demand decreased the most in the lower stratum for concentrated SSBs only [27].

We used the SANHANES-1 to estimate baseline consumption of different drinks. However, comparison to soft drink sales suggests that our estimates may have underestimated consumption. Coca Cola company figures show that average per capita consumption of Coca Cola products is 67 litres of soft drinks a year in SA, or 184 ml a day [59] whereas our consumption estimates were 184 ml of all SSBs per day including concentrates, carbonated drinks and sweetened fruit juice. Coca Cola products account for about half of the soft drinks market [66] so the average South African is potentially consuming about 400 ml of SSBs daily.

In the UK study by Briggs et al., sales figures reported 446 ml/person/day compared to the 123 ml/person/day used in their model [27]. For the purpose of calculating the model consumption inputs the maximum and minimum amount of milk and unsweetened fruit juice were capped at 500 ml and 125 ml respectively. The potential impact of capping the maximum at 500 ml would be underestimating consumption and capping the minimum amount would possibly result in overestimating consumption. Furthermore, the SANHANES-1 consumption estimates were self-reported and may have been affected by recall bias. We assumed that all milk drunk was full cream milk because the SANHANES-1 data did not have details of types of milk. This may have overestimated the caloric shift due to substitution effect.

We did not explicitly account for substitution of SSBs with other sugar-sweetened food items. We assumed that SSBs would be substituted with other drinks and other studies show that substitution of drinks with other foods is insignificant [64].

An increase in the price of SSBs may lead to a reduction in food waste and hence not result in the projected increased consumption. This would reduce the change in BMI and obesity levels. SSBs are however easy to keep and don't easily rot or go stale. There is lack of evidence of exactly how much of the SSBs and other food is wasted or the price elasticity of food waste in LMICs but it is known that in high income countries substantial waste occurs at household level [67]. Another consequence of increased SSB price may be price shocks where changes in purchasing are due to a signal that SSBs are unhealthy and are therefore being taxed rather than the price change itself, and this may be more than what we modelled. A 100% pass on rate was assumed but the tax increase may not be passed on in full. However, our sensitivity analysis shows that with 20% tax rate and pass on rates ranging from 80–120%, the estimates of the effect on obesity shift by no more than 0.6 percentage points. Data from France also showed that a pass on rate of 100% is plausible [48].

Our model compares the current situation with a counterfactual situation in which higher SSB prices reduce consumption and individuals lose weight until they are in a new equilibrium, given their energy consumption and assuming stable levels of physical activity. It does not capture a possible effect of the intervention on the rate of weight gain over time that is caused by a small but persistent average daily energy imbalance. It is therefore possible that our study, and all previous studies that made the same assumption [28], [29], underestimate the cumulative impact of changes in SSB consumption on body mass over the years.

### Policy implications

The DOH considers fiscal policy to be a cost effective ‘best buy’ that can be used to prevent obesity and NCDs [39]. This study provides evidence in the SA context of how one such fiscal policy would impact obesity. Such a policy would form part of a multi-faceted and intersectorial approach to tackling NCDs in SA [39]. Other interventions that the DOH has listed as ‘best buys’ in NCD prevention include promotion of physical exercise and creating an environment conducive to exercising, increased health education and early detection of NCDs [39].

The SA Declaration on the Prevention and Control of NCDs signed in 2011 includes a commitment to “reduce by 10% the percentage of people who are obese and/or overweight by 2020” [39]. By reducing obesity in adults by 2.8%, an SSB tax would potentially contribute towards approximately 25% of this target and the reduction would even be greater by 2020. Studies show that half the weight change can be achieved in one year and 95% in three years [68].

The SSB tax would also contribute towards relieving pressure on an already fragile health system. The tax would contribute substantially to the multi-sectorial and ‘whole of government’ and ‘whole of society’ approach envisaged by the DOH.

Taxing SSBs can potentially raise considerable revenue. An SSB tax of a penny per ounce in the state of Louisiana, USA, would have generated approximately 210 million USD in 2013, equivalent to over two billion Rands at current rates [69]. These funds can be dedicated in part or wholly to public health programs including subsidising fruits and vegetables, social marketing campaigns, school-based nutrition programs and enhancing the built environment to increase physical activity. Polls conducted in the USA between 2001 and 2004 show that the public favoured an SSB tax if the revenue were to be used to support child nutrition programs [70]. A 2008 poll of New York State residents showed that 52% of respondents supported a soda tax and that 72% supported such a tax if the revenue was used to support programs for the prevention of obesity in children and adults [71].

As our study and others have shown, an increase in the price of SSBs will result in reduced purchasing and consumption of SSBs. This may increase the demand for healthier alternatives and influence manufacturers to reformulate their products [69]. The SSB tax may disproportionately affect the poor [69]. However the poor are also disproportionately affected by obesity and NCDs, have less access to healthcare and less income available to seek healthcare. The tax would potentially reduce these health inequities.

### Recommendations for further research

To characterise the potential cumulative effects of an SSB tax over time, the possible effect of SSB consumption on the daily energy balance and weight change should be investigated. However, this may require a degree of measurement of energy intake and expenditure that is currently not achievable.

The consumption levels of SSBs in SA have not been sufficiently quantified. Further work is required to determine consumption levels and trends in both adults and children. The possible impact of projected increases in soft drink sales on obesity also needs to be investigated to better understand future gains of the tax.

Further research is needed to determine the extent to which reduced SSB sales would have adverse economic or social consequences such as job losses, and whether these would outweigh the benefits of reduced obesity and its related diseases, increased productivity and quality of life. A study conducted in the states of Illinois and California in the USA showed that SSB taxes did not have a negative impact on state-level employment. This macroeconomic simulation study found that declines in the beverage industry occurred but were offset by new employment in non-beverage industry and government sectors [72].

The role of public opinion in the uptake of fiscal policy is important and has not been explored. The SA public's nutritional literacy in relation to SSBs, their understanding of the role of sugar in obesity, and their perception of the benefits and drawbacks of an SSB tax needs to be assessed. Further research is also needed to quantify the impact of taxing SSBs on the burden of obesity-related NCDs and on life expectancy.

## Supporting Information

Figure S1 **Comparison of raw and lognormal data and a counterfactual distribution for mean BMI in males.** A comparison of the raw mean BMI data (data) with the log-normal fitted BMI data (lognorm) and counterfactual log-normal data (counterfactual) in which mean BMI was increased by five BMI points, for males (M) by age-group.
(PDF)

Figure S2 **Comparison of raw and lognormal data and a counterfactual distribution for mean BMI in females.** A comparison of the raw mean BMI data (data) with the log-normal fitted BMI data (lognorm) and counterfactual log-normal data (counterfactual) in which mean BMI was increased by five BMI points, for females (F) by age-group.
(PDF)

Table S1 **Comparison of the log-normal and gamma distributions for the NIDS Wave 3 BMI data.** Comparison of measures of fit (Residuals and Difference in mean) between log-normal and gamma fitted BMI data.
(DOCX)

Text S1 **Fitting BMI data to the lognormal distribution.** A description of fitting the BMI data to the lognormal distribution and comparison between the gamma and lognormal distributions.
(DOCX)

## References

[1] Popkin BM, Adair LS, Ng SW (2012) Global nutrition transition and the pandemic of obesity in developing countries. *Nutr Rev* 70: 3–21. 22221213 10.1111/j.1753-4887.2011.00456.x PMC3257829

[2] Finucane MM, Stevens GA, Cowan MJ, Danaei G, Lin JK, et al (2011) National, regional, and global trends in body-mass index since 1980: systematic analysis of health examination surveys and epidemiological studies with 960 country-years and 9·1 million participants. *Lancet* 377: 557–567. 21295846 10.1016/S0140-6736(10)62037-5 PMC4472365

[3] Popkin BM, Slining MM (2013) New dynamics in global obesity facing low- and middle-income countries. *Obes Rev (Suppl 2)* 14: 11–20. 10.1111/obr.12102 PMC4074506 24102717

[4] Department of Health MRC (2007) South Africa Demographic and Health Survey 2003. Pretoria: Department of Health. 258–297 p.Available: http://www.mrc.ac.za/bod/sadhs2003fullreport.pdf. Accessed: 25 November 2013

[5] Shisana O, Labadarios D, Rehle T, Simbayi L, Zuma K, et al. (2013) South African National Health and Nutrition Examination Survey (SANHANES-1). Cape Town HSRC Press. 135-144 p.Available: http://www.hsrc.ac.za/uploads/pageNews/72/SANHANES-launch%20edition%20(online%20version).pdf. Accessed 19 November 2013.

[6] Ng M, Fleming T, Robinson M, Thomson B, Graetz N, et al.. (2014) Global, regional, and national prevalence of overweight and obesity in children and adults during 1980–2013: a systematic analysis for the Global Burden of Disease Study 2013. The Lancet. 10.1016/S0140-6736(14)60460-8 PMC4624264 24880830

[7] Joubert J, Norman R, Bradshaw D, Goedecke JH, Steyn NP, et al (2007) Estimating the burden of disease attributable to excess body weight in South Africa in 2000. *S Afr Med J* 97: 683–690. 17952225

[8] Malaza A, Mossong J, Bärnighausen T, Newell M-L (2012) Hypertension and obesity in adults living in a high HIV prevalence rural area in South Africa. *PloS One* 7: e47761. 23082211 10.1371/journal.pone.0047761 PMC3474786

[9] WHO (2009) Global health risks: mortality and burden of disease attributable to selected major risks. Geneva: WHO Available: http://www.who.int/healthinfo/global_burden_disease/GlobalHealthRisks_report_full.pdf. Accessed 19 November 2013

[10] Murray CJ, Vos T, Lozano R, Naghavi M, Flaxman AD, et al (2013) Disability-adjusted life years (DALYs) for 291 diseases and injuries in 21 regions, 1990–2010: a systematic analysis for the Global Burden of Disease Study 2010. The Lancet 380: 2197–2223. 10.1016/S0140-6736(12)61689-4 23245608

[11] Mayosi BM, Flisher AJ, Lalloo UG, Sitas F, Tollman SM, et al (2009) The burden of non-communicable diseases in South Africa. *Lancet* 374: 934–947. 19709736 10.1016/S0140-6736(09)61087-4

[12] Bertram MY, Jaswal AV, Van Wyk VP, Levitt NS, Hofman KJ (2013) The non-fatal disease burden caused by type 2 diabetes in South Africa, 2009. *Global Health Action* 6: 19244. 23364089 10.3402/gha.v6i0.19244 PMC3556685

[13] Lengerke Tv, Krauth C (2011) Economic costs of adult obesity: A review of recent European studies with a focus on subgroup-specific costs. *Maturitas* 69: 220–229. 21592692 10.1016/j.maturitas.2011.04.005

[14] Lobstein T (2011) Prevalence and costs of obesity. *Medicine* 39: 11–13.

[15] Cawley J, Meyerhoefer C (2012) The medical care costs of obesity:An instrumental variables approach. *J Health Econ* 31: 219–230. 22094013 10.1016/j.jhealeco.2011.10.003

[16] Sturm R, An R, Maroba J, Patel D (2013) The effects of obesity, smoking, and excessive alcohol intake on healthcare expenditure in a comprehensive medical scheme. *S Afr Med J* 103: 840–844. 24148168 10.7196/SAMJ.7260 PMC3807241

[17] Bertram MY, Katzenellenbogen J, Vos T, Bradshaw D, Hofman KJ (2013) The disability adjusted life years due to stroke in South Africa in 2008. *Int J Stroke* 8: 76–80. 23295022 10.1111/j.1747-4949.2012.00955.x

[18] Ebbeling CB, Feldman HA, Osganian SK, Chomitz VR, Ellenbogen SJ, et al (2006) Effects of Decreasing Sugar-Sweetened Beverage Consumption on Body Weight in Adolescents: A Randomized, Controlled Pilot Study. *Pediatrics* 117: 673–680. 16510646 10.1542/peds.2005-0983

[19] Malik VS, Schulze MB, Hu FB (2006) Intake of sugar-sweetened beverages and weight gain: a systematic review. *Am J Clin Nutr* 84: 274–288. 16895873 10.1093/ajcn/84.1.274 PMC3210834

[20] Hu FB, Malik VS (2010) Sugar-sweetened beverages and risk of obesity and type 2 diabetes: epidemiologic evidence. *Physiol Behav* 100: 47–54. 20138901 10.1016/j.physbeh.2010.01.036 PMC2862460

[21] Malik VS, Popkin BM, Bray GA, Despres JP, Willett WC, et al (2010) Sugar-sweetened beverages and risk of metabolic syndrome and type 2 diabetes: a meta-analysis. *Diabetes Care* 33: 2477–2483. 20693348 10.2337/dc10-1079 PMC2963518

[22] de Ruyter JC, Olthof MR, Seidell JC, Katan MB (2012) A trial of sugar-free or sugar-sweetened beverages and body weight in children. *N Engl J Med* 367: 1397–1406. 22998340 10.1056/NEJMoa1203034

[23] Chen L, Appel LJ, Loria C, Lin P-H, Champagne CM, et al (2009) Reduction in consumption of sugar-sweetened beverages is associated with weight loss: the PREMIER trial. *Am J Clin Nutr* 89: 1299–1306. 19339405 10.3945/ajcn.2008.27240 PMC2676995

[24] Malik VS, Popkin BM, Bray GA, Després J-P, Hu FB (2010) Sugar-sweetened beverages, obesity, type 2 diabetes mellitus, and cardiovascular disease risk. Circulation 121: 1356–1364. 20308626 10.1161/CIRCULATIONAHA.109.876185 PMC2862465

[25] Warren JJ, Weber-Gasparoni K, Marshall TA, Drake DR, Dehkordi-Vakil F, et al (2009) A longitudinal study of dental caries risk among very young low SES children. Community dentistry and oral epidemiology 37: 116–122. 19046332 10.1111/j.1600-0528.2008.00447.x PMC2661009

[26] Armfield JM, Spencer AJ, Roberts-Thomson KF, Plastow K (2013) Water fluoridation and the association of sugar-sweetened beverage consumption and dental caries in Australian children. American journal of public health 103: 494–500. 23327241 10.2105/AJPH.2012.300889 PMC3673496

[27] Briggs AD, Mytton OT, Kehlbacher A, Tiffin R, Rayner M, et al (2013) Overall and income specific effect on prevalence of overweight and obesity of 20% sugar sweetened drink tax in UK: econometric and comparative risk assessment modelling study. *BMJ* 347: f6189. 24179043 10.1136/bmj.f6189 PMC3814405

[28] Briggs AD, Mytton OT, Madden D, O'Shea D, Rayner M, et al (2013) The potential impact on obesity of a 10% tax on sugar-sweetened beverages in Ireland, an effect assessment modelling study. *BMC Public Health* 13: 860. 24044370 10.1186/1471-2458-13-860 PMC3852031

[29] Claro RM, Levy RB, Popkin BM, Monteiro CA (2012) Sugar-sweetened beverage taxes in Brazil. *Am J Public Health* 102: 178–183. 22095333 10.2105/AJPH.2011.300313 PMC3490548

[30] Cabrera Escobar MA, Veerman JL, Tollman SM, Bertram MY, Hofman KJ (2013) Evidence that a tax on sugar sweetened beverages reduces the obesity rate: a meta-analysis. *BMC Public Health* 13: 1072. 24225016 10.1186/1471-2458-13-1072 PMC3840583

[31] Basu S, Vellakkal S, Agrawal S, Stuckler D, Popkin B, et al (2014) Averting Obesity and Type 2 Diabetes in India through Sugar-Sweetened Beverage Taxation: An Economic-Epidemiologic Modeling Study. *PLoS medicine* 11: e1001582. 24409102 10.1371/journal.pmed.1001582 PMC3883641

[32] EU Food Law (2013) *Denmark drops decades old soft drinks tax* England Informa UK PLC. Available: http://www.eurofoodlaw.com/country-reports/eu-member-states/denmark/denmark-drops-decades-old-soft-drinks-tax-64140.htm. 09 January.

[33] Cheney C (2011) *Battling the Couch Potatoes: Hungary Introduces 'Fat Tax'*. Spiegel Online International: Spiegel Online.

[34] Spiegel (2011) *French 'Cola Tax' Approved: Paris Vows to Fight Deficit and Obesity*. Spiegel Online International. England: Spiegel Online.

[35] UNESDA (2010) *Why discriminatory taxes don't work* Brussels UNESDA Available: http://www.unesda.org/why-discriminatory-taxes-don-t-work#topofall. 9 January.

[36] Chattan N (2013) *Mexican Congress Passes Tax Bill With Higher Junk Food Levy*. Bloomberg Personal Finance. New York City: Bloomberg L.P.

[37] Van Walbeek C (2003) Tobacco excise taxation in South Africa. Geneva: World Health Organisation. Available: http://www.who.int/tobacco/training/success_stories/en/best_practices_south_africa_taxation.pdf. Accessed 26 November 2013.

[38] De Wet P (2011) *D-Day for heart-attack fats, but don't touch me on my full-cream milk*. Daily Maverick. Johannesburg: Styli Charalambous.

[39] Department of Health Strategic Plan for the Prevention and Control of Non-Communicable Diseases 2013–17. Johannesburg: National Department of Health. 26–44 p.Available: http://www.hsrc.ac.za/uploads/pageContent/3893/NCDs%20STRAT%20PLAN%20%20CONTENT%208%20april%20proof.pdf. Accessed 27 November 2013.

[40] Holmes T (2013) *Salt sellers shaken by Motsoaledi's rules*. Mail and Guardian. Johannesburg: M&G Media Limited.

[41] Dodds C (2013) *Minister of Health goes sour on sugar*. Saturday Star. Johannesburg: Independent Newspapers.

[42] Swinburn BA, Sacks G, Lo SK, Westerterp KR, Rush EC, et al (2009) Estimating the changes in energy flux that characterize the rise in obesity prevalence. *Am J Clin Nutr* 89: 1723–1728. 19369382 10.3945/ajcn.2008.27061 PMC3738432

[43] Veerman JL, Van Beeck EF, Barendregt JJ, Mackenbach JP (2009) By how much would limiting TV food advertising reduce childhood obesity? *Eur J Public Health* 19: 365–369. 19324935 10.1093/eurpub/ckp039 PMC2712920

[44] Andreyeva T, Chaloupka FJ, Brownell KD (2011) Estimating the potential of taxes on sugar-sweetened beverages to reduce consumption and generate revenue. *Prev Med* 52: 413–416. 21443899 10.1016/j.ypmed.2011.03.013

[45] Sacks G, Veerman JL, Moodie M, Swinburn B (2011) 'Traffic-light' nutrition labelling and 'junk-food' tax: a modelled comparison of cost-effectiveness for obesity prevention. *Int J Obes* 35: 1001–1009. 10.1038/ijo.2010.228 21079620

[46] Cabrera-Escobar MA, Veerman LJ, Tollman S, Bertram M, Hofman K (2013) Evidence that a tax on sugar-sweetened beverages reduces the obesity rate-meta analysis. *BMC Public Health* 13. 10.1186/1471-2458-13-1072 PMC3840583 24225016

[47] Briggs AD, Mytton OT, Madden D, O'Shea D, Rayner M, et al.. (2013) The potential impact on obesity of a 10% tax on sugar-sweetened beverages in Ireland, an effect assessment modelling study. BMC Public Health 13. 10.1186/1471-2458-13-860 PMC3852031 24044370

[48] Berardi N, Sevestere P, Tepaut M, Vigneron A (2012) The Impact of a "Soda Tax" on prices: Evidence from French microdata. *Banque de France Working Paper* 415.

[49] Kenkel D (2005) Are alcohol tax hikes fully passed through to prices? Evidence from Alaska. *Am Econ Rev* 95: 273–277.

[50] NIDS (2013) National Income Dynamics Study 2012, Wave 3 SALDRU, Capetown: DataFirst. 1.0. Available: https://www.datafirst.uct.ac.za/dataportal/index.php/catalog/453. Accessed 19 November 2013.

[51] Leibbtandt M, Woolard I, de Villiers L (2009) Methodology: Report on NIDS Wave 1, Technical Paper no. 1. SALDRU. Available: http://www.nids.uct.ac.za/home/index.php?/Nids-Documentation/technical-papers.html. Accessed 16 July 2013.

[52] Brown M, Daniels R.C., De Villiers, L., Leibbrandt, , &Woolard, I, ., eds. (2012) National Income Dynamics Study Wave 2 User Manual-Updated. Capetown: SALDRU. Available: http://www.nids.uct.ac.za/documents/wave-2-documents-and-questionnaires/198-nids-wave-2-user-manual-updated. Accessed 19 November 2013.

[53] De Villiers L, Brown M., Woolard I., Daniels R.C., & Leibbrandt M, eds. (2013) National Income Dynamics Study Wave 3 User Manual. Capetown: SALDRU.Available: http://www.nids.uct.ac.za/documents/157-nids-wave-3-user-manual?path=. Accessed 19 November 2013.

[54] NIDS (2012) Wave 1 Update: May 2012, Introduction to NIDS Data. Capetown: SALDRU. Available: http://www.nids.uct.ac.za/home/index.php?/Nids-Documentation/documents.html. Accessed 15 July 2013.

[55] NIDS (2008) Fieldwork Manual Wave 1. Capetown: SALDRU. Available: http://www.nids.uct.ac.za/home/index.php?/Nids-Documentation/documents.html. Accessed 16 July 2013.

[56] STATA (2011) Stata statistical software: Release 12. 12.1 ed. College Station, TX: StataCorpLP.

[57] Goris JM, Petersen S, Stamatakis E, Veerman JL (2010) Television food advertising and the prevalence of childhood overweight and obesity: a multicountry comparison. Public health nutrition 13: 1003–1012. 20018123 10.1017/S1368980009992850

[58] Veerman JL, Barendregt JJ, Beeck EF, Seidell JC, Mackenbach JP (2007) Stemming the obesity epidemic: a tantalizing prospect. Obesity 15: 2365–2370. 17890506 10.1038/oby.2007.280

[59] Adami N, Ustas J, Penhale I, Leibowitz G (2012) Quarterly divisional seminar series South Africa. London: SABMiller. Available: http://www.sabmiller.com/files/presentations/2009/220909/220909_africaasia_quarterly_seminar.pdf. Accessed 4 December 2014.

[60] StatsSA (2013) Mid-year population estimates 2013. Pretoria: StatsSA.Available: http://www.statssa.gov.za/publications/SAStatistics/SAStatistics2013.pdf. Accessed 19 November 2013.

[61] Parmalat (2011) Everfresh Milk nutritional information per 100ml. Johannesburg Parmalat. Available: http://www.parmalat.co.za/index.php?id=15, Accessed 14 February 2014.

[62] Rose G, Day S (1990) The population mean predicts the number of deviant individuals. *BMJ* 301: 1031–1034. 2249053 10.1136/bmj.301.6759.1031 PMC1664038

[63] Southern Africa Labour and Development Research Unit (2014) South Africa - National Income Dynamics Study 2012, Wave 3. Cape Town: SALDRU.Available: http://www.nids.uct.ac.za/documents/wave-3-documents-and-questionnaires/190-nids-wave-3-overview-document. Accessed 24 March 2014.

[64] Finkelstein EA, Zhen C, Bilger M, Nonnemaker J, Farooqui AM, et al (2013) Implications of a sugar-sweetened beverage (SSB) tax when substitutions to non-beverage items are considered. *J Health Econ* 32: 219–239. 23202266 10.1016/j.jhealeco.2012.10.005

[65] Finkelstein EA, Zhen C, Nonnemaker J, Todd JE (2010) Impact of targeted beverage taxes on higher-and lower-income households. Archives of Internal Medicine 170: 2028–2034. 21149762 10.1001/archinternmed.2010.449

[66] Euromonitor International (2013) Soft Drinks in South Africa: Industry Overview United Kingdom: Euromonitor International. Available: http://0-researchmonitor.euromonitor.com.innopac.wits.ac.za/. Accessed 21 February 2014.

[67] Godfray HCJ, Crute IR, Haddad L, Lawrence D, Muir JF, et al (2010) The future of the global food system. Philosophical Transactions of the Royal Society B: Biological Sciences 365: 2769–2777. 10.1098/rstb.2010.0180 PMC2935131 20713383

[68] Hall KD, Sacks G, Chandramohan D, Chow CC, Wang YC, et al (2011) Quantification of the effect of energy imbalance on bodyweight. The Lancet 378: 826–837. 10.1016/S0140-6736(11)60812-X PMC3880593 21872751

[69] Roberta R, Friedman, Kelly D Brownell (2012) Sugar-sweetened beverage taxes: An updated policy brief. Yale Rudd Centre for Food Policy and Obesity. 1–8 p.Available: http://www.yaleruddcenter.org/resources/upload/docs/what/reports/Rudd_Policy_Brief_Sugar_Sweetened_Beverage_Taxes.pdf. Accessed 24 March 2014.

[70] Brownell KD (2005) The chronicling of obesity: growing awareness of its social, economic, and political contexts. Journal of Health Politics, Policy and Law 30: 955–964. 10.1215/03616878-30-5-955 16477793

[71] Brownell KD, Farley T, Willett WC, Popkin BM, Chaloupka FJ, et al (2009) The public health and economic benefits of taxing sugar-sweetened beverages. *N Engl J Med* 361: 1599–1605. 19759377 10.1056/NEJMhpr0905723 PMC3140416

[72] Powell LM, Wada R, Persky JJ, Chaloupka FJ (2014) Employment impact of sugar-sweetened beverage taxes. Am J Public Health 104: 672–677. 24524492 10.2105/AJPH.2013.301630 PMC4025719

