# Supplementary material for: The Potential Impact of a 20% Tax on Sugar-Sweetened Beverages on Obesity in South African Adults: A Mathematical Model
Source: PLoS One. 2014 Aug 19;9(8):e105287. doi: 10.1371/journal.pone.0105287 (PMC4138175; doi:10.1371/journal.pone.0105287)
Supplement: Figure S1 — Comparison of raw and lognormal data and a counterfactual distribution for mean BMI in males. A comparison of the raw mean BMI data (data) with the log-normal fitted BMI data (lognorm) and counterfactual log-normal data (counterfactual) in which mean BMI was increased by five BMI points, for males (M) by age-group. (PDF) [file pone.0105287.s001.pdf]

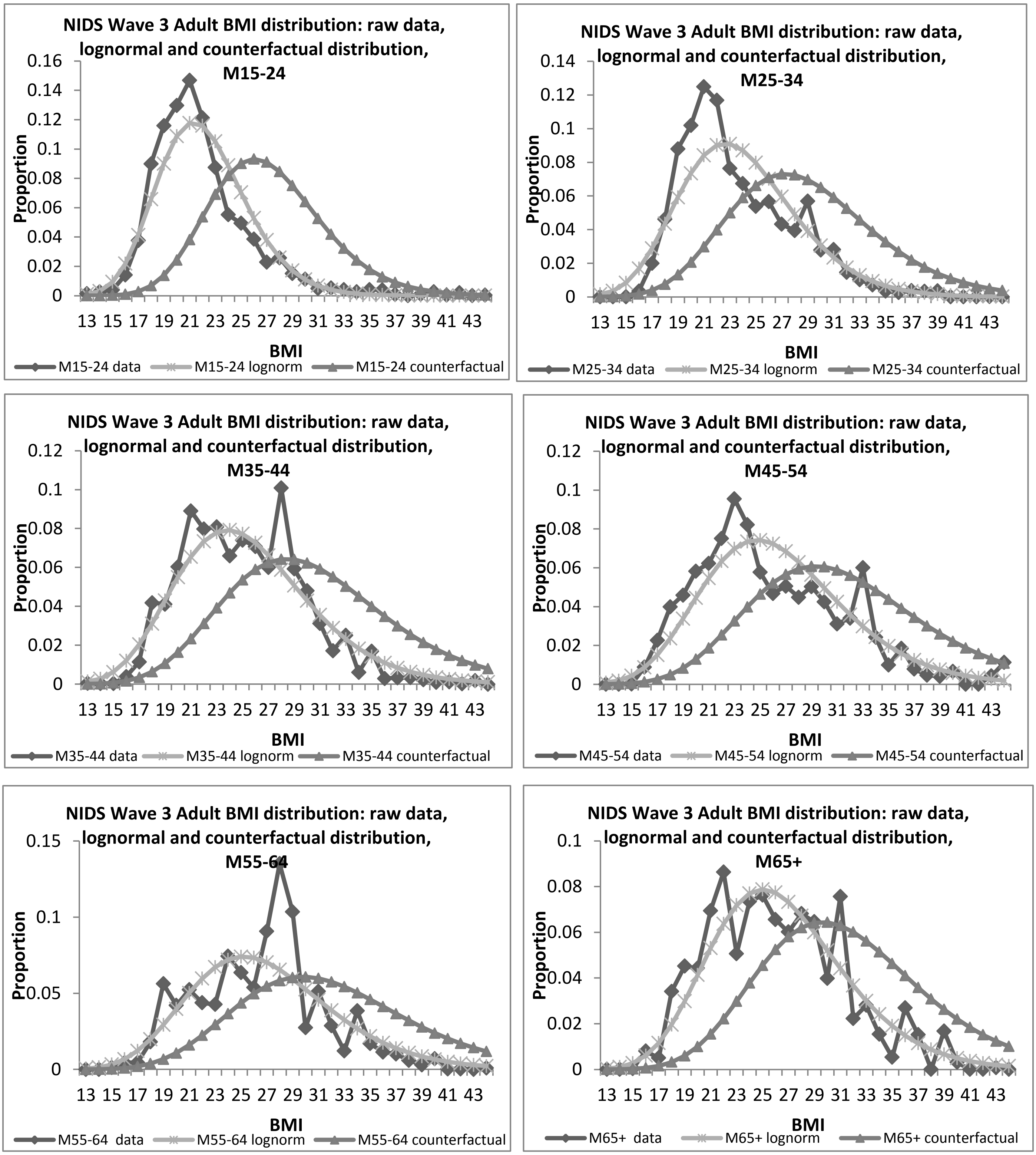

Figure S1 Comparison of raw and lognormal data and a counterfactual distribution for mean BMI in males
