# Supplementary material for: The Potential Impact of a 20% Tax on Sugar-Sweetened Beverages on Obesity in South African Adults: A Mathematical Model
Source: PLoS One. 2014 Aug 19;9(8):e105287. doi: 10.1371/journal.pone.0105287 (PMC4138175; doi:10.1371/journal.pone.0105287)
Supplement: Table S1 — Comparison of the log-normal and gamma distributions for the NIDS Wave 3 BMI data. Comparison of measures of fit (Residuals and Difference in mean) between log-normal and gamma fitted BMI data. (DOCX) [file pone.0105287.s003.docx]

Table S1 Comparison of log-normal and gamma distributions for the NIDS Wave 3 data

|  | **Residuals** | |  | **Means** | | | **Difference in mean** | |
| --- | --- | --- | --- | --- | --- | --- | --- | --- |
| **Age** | **Log-normal** | **Gamma** | **Difference** | **Data** | **Log-normal** | **Gamma** | **Log-normal** | **Gamma** |
| **Male** |  |  |  |  |  |  |  |  |
| **15-24** | 0.0008 | 0.0010 | 0.0002 | 21.93 | 22.27 | 22.16 | 0.34 | 0.24 |
| **25-34** | 0.0020 | 0.0022 | 0.0002 | 23.63 | 24.48 | 24.28 | 0.85 | 0.64 |
| **35-44** | 0.0033 | 0.0032 | -0.0001 | 25.11 | 25.72 | 25.79 | 0.60 | 0.68 |
| **45-54** | 0.0027 | 0.0026 | -0.0001 | 25.87 | 26.51 | 26.56 | 0.65 | 0.69 |
| **55-64** | 0.0103 | 0.0094 | -0.0009 | 26.55 | 27.69 | 26.56 | 1.14 | 0.01 |
| **65+** | 0.0036 | 0.0036 | -0.0001 | 26.09 | 26.63 | 27.14 | 0.55 | 1.06 |
| **Average** | 0.0038 | 0.0037 | -0.0001 | 24.86 | 25.55 | 25.42 | 0.69 | 0.55 |
|  |  |  |  |  |  |  |  |  |
| **Female** |  |  |  |  |  |  |  |  |
| **15-24** | 0.0007 | 0.0008 | 0.0001 | 24.22 | 24.71 | 24.58 | 0.49 | 0.36 |
| **25-34** | 0.0017 | 0.0016 | -0.0001 | 27.73 | 28.17 | 28.28 | 0.43 | 0.55 |
| **35-44** | 0.0021 | 0.0019 | -0.0001 | 29.52 | 29.79 | 29.65 | 0.27 | 0.13 |
| **45-54** | 0.0025 | 0.0022 | -0.0003 | 29.98 | 30.44 | 30.15 | 0.47 | 0.17 |
| **55-64** | 0.0076 | 0.0056 | -0.0019 | 30.34 | 31.19 | 30.15 | 0.86 | -0.19 |
| **65+** | 0.0060 | 0.0054 | -0.0006 | 28.76 | 29.66 | 30.84 | 0.89 | 2.08 |
|  |  |  |  |  |  |  |  |  |
| **Average** | 0.0034 | 0.0029 | -0.0005 | 28.42 | 28.99 | 28.94 | 0.57 | 0.52 |
