# Supplementary material for: The Potential Impact of a 20% Tax on Sugar-Sweetened Beverages on Obesity in South African Adults: A Mathematical Model
Source: PLoS One. 2014 Aug 19;9(8):e105287. doi: 10.1371/journal.pone.0105287 (PMC4138175; doi:10.1371/journal.pone.0105287)
Supplement: Text S1 — Fitting BMI data to the lognormal distribution. A description of fitting the BMI data to the lognormal distribution and comparison between the gamma and lognormal distributions. (DOCX) [file pone.0105287.s004.docx]

# Text S1

## Fitting BMI data to the lognormal distribution

### BMI Frequencies

Using the Stata *svy, subpop* command, frequencies for each BMI unit were computed for males and females for each of the ten-year age-groups and these were copied into Microsoft Excel 2010. The BMI ranged from 13 to 44 for all ages and both sexes.

### Fitting in Microsoft Excel

The *lognormdist* function in Excel was used to transform the BMI frequencies to the lognormal distribution. We also modelled a counterfactual scenario of an increase in mean BMI of five BMI units to demonstrate how the BMI distribution would respond with changes in mean BMI. Figures S1 and S2 shows the raw data compared to the lognormal data, and the counterfactual distribution.

The residuals between the raw data and the lognormal distribution for the single BMI units and for the mean BMI for each age group were squared and summed. The parameters µ and σ for the lognormal function in Excel were calculated using the following equations:

µ = ln (mean) - σ^2^/2

σ=√ln(SD^2^)/mean^2^+1, where mean and SD are the mean and standard deviation of the fitted lognormal distributions.

To ensure the distributions changed gradually across the ages we removed (stochastic) variation. Quadratic functions were incorporated to fit the means and standard deviations across age-groups for each sex. The sum of residual squares were minimised using the solver function in Excel with constraints on the constants for the quadratic functions. The quadratic functions were as follows:

For means: a+bx+cx^2^+dx^3^

For standard deviations: a+bx+cx^2^+x

### Comparison of log-normal and gamma distributions

We copied the frequencies for each BMI unit were for males and females for each of the ten-year age-groups into Microsoft Excel 2010. These data were fit to the gamma distribution with parameters α and β and an intercept. The sum of the squared residual was minimised using the Excel 2010 solver function with constraints on the parameters and the intercept. The data were similarly fitted to the log-normal distribution with parameters µ and σ and an intercept, and the solver function used to minimise the sum of squared residuals. Table S1 shows the results of the comparison between the log-normal and the gamma fit. The results in Table S1 show although the gamma distribution seems provide a slightly better fit than the lognormal distribution, the residuals and mean BMI values are largely similar. The gamma distribution did not lend itself well to our fitting procedure described above and therefore we used the log-normal for our model.
